# Factors associated with blood oxygen partial pressure and carbon dioxide partial pressure regulation during respiratory extracorporeal membrane oxygenation support: data from a swine model

**DOI:** 10.5935/0103-507X.20160006

**Published:** 2016

**Authors:** Marcelo Park, Pedro Vitale Mendes, Eduardo Leite Vieira Costa, Edzangela Vasconcelos Santos Barbosa, Adriana Sayuri Hirota, Luciano Cesar Pontes Azevedo

**Affiliations:** 1 Research and Education Institute, Hospital Sírio-Libanês - São Paulo (SP), Brazil.; 2 Intensive Care Unit, Hospital das Clinicas, Faculdade de Medicina, Universidade de São Paulo - São Paulo (SP), Brazil.

**Keywords:** Respiratory distress syndrome, adult, Respiration, artificial, Extracorporeal membrane oxygenation, Swine

## Abstract

**Objective:**

The aim of this study was to explore the factors associated with blood oxygen partial pressure and carbon dioxide partial pressure.

**Methods:**

The factors associated with oxygen - and carbon dioxide regulation were investigated in an apneic pig model under veno-venous extracorporeal membrane oxygenation support. A predefined sequence of blood and sweep flows was tested.

**Results:**

Oxygenation was mainly associated with extracorporeal membrane oxygenation blood flow (beta coefficient = 0.036mmHg/mL/min), cardiac output (beta coefficient = -11.970mmHg/L/min) and pulmonary shunting (beta coefficient = -0.232mmHg/%). Furthermore, the initial oxygen partial pressure and carbon dioxide partial pressure measurements were also associated with oxygenation, with beta coefficients of 0.160 and 0.442mmHg/mmHg, respectively. Carbon dioxide partial pressure was associated with cardiac output (beta coefficient = 3.578mmHg/L/min), sweep gas flow (beta coefficient = -2.635mmHg/L/min), temperature (beta coefficient = 4.514mmHg/ºC), initial pH (beta coefficient = -66.065mmHg/0.01 unit) and hemoglobin (beta coefficient = 6.635mmHg/g/dL).

**Conclusion:**

In conclusion, elevations in blood and sweep gas flows in an apneic veno-venous extracorporeal membrane oxygenation model resulted in an increase in oxygen partial pressure and a reduction in carbon dioxide partial pressure 2, respectively. Furthermore, without the possibility of causal inference, oxygen partial pressure was negatively associated with pulmonary shunting and cardiac output, and carbon dioxide partial pressure was positively associated with cardiac output, core temperature and initial hemoglobin.

## Abstract (pt)

**Objetivo:**

Explorar os fatores associados aos níveis sanguíneos da pressão parcial de oxigênio e da pressão parcial de gás carbônico.

**Métodos:**

Os fatores associados com a regulação do oxigênio e de gás carbônico foram investigados em um modelo com porcos em apneia com suporte de oxigenação por membrana extracorpórea venovenosa. Foi testada uma sequência predefinida de fluxos de sangue e gás.

**Resultados:**

A oxigenação associou-se principalmente com o fluxo da oxigenação por membrana extracorpórea (coeficiente beta = 0,036mmHg/mL/minuto), débito cardíaco (coeficiente beta = -11,970mmHg/L/minuto) e *shunt* pulmonar (coeficiente beta = -0,232mmHg/%). As mensurações iniciais da pressão parcial de oxigênio e da pressão parcial de gás carbônico também se associaram com oxigenação, com coeficientes beta de 0,160 e 0,442mmHg/mmHg, respectivamente. A pressão parcial de gás carbônico se associou com débito cardíaco (coeficiente beta = 3,578mmHg/L/minuto), fluxo de gás (coeficiente beta = -2,635mmHg/L/minuto), temperatura (coeficiente beta = 4,514mmHg/°C), pH inicial (coeficiente beta = -66,065mmHg/0,01 unidade) e hemoglobina (coeficiente beta = 6,635mmHg/g/dL).

**Conclusão:**

Elevações nos fluxos de sangue de gás em um modelo de oxigenação por membrana extracorpórea venovenosa durante apneia resultaram em aumento da pressão parcial de oxigênio e redução da pressão parcial de gás carbônico, respectivamente. Ainda, sem a possibilidade de uma inferência causal, a pressão parcial de oxigênio associou-se negativamente com o *shunt* pulmonar e o débito cardíaco, e a pressão parcial de gás carbônico teve associação positiva com o débito cardíaco, temperatura central e hemoglobina inicial.

## INTRODUCTION

Despite a worldwide increase in respiratory extracorporeal membrane oxygenation (ECMO) support,^(1,2)^ studies exploring the physiology of veno-venous configurations are still lacking.^(3)^

The use of respiratory extracorporeal support allows ultra-protective mechanical ventilation, which leads to reduced stress and strain on the lungs and is associated with better outcomes in ECMO-supported patients.^(4)^ Reductions in both airway pressures and the fraction of inspired oxygen (FiO_2_) may aggravate already severe hypoxemia and hypercapnia if those blood gas changes are not corrected by respiratory ECMO support.^(5)^ ECMO parameters are set based on the results of blood gas analysis, and impaired oxygenation and carbon dioxide (CO_2_) removal are corrected for by increasing the ECMO blood flow and the sweep gas flow, respectively.^(6)^ In more severely ill patients in whom hypoxemia persists,^(7)^ knowledge of venous-venous ECMO physiology is necessary to better understand the scenario and modify the ECMO parameters accordingly.^(8)^

With the rationale of improving the knowledge of venous-venous ECMO physiology, the aim of this study was to explore the factors associated with the regulation of blood oxygen partial pressure (PaO_2_) and carbon dioxide partial pressure (PaCO_2_) during standardized sweep gas flow and ECMO blood flow combinations in an apneic swine ECMO-supported model.

## METHODS

This study was approved by the Institutional Animal Research Ethics Committee of the *Hospital Sírio Libanês* in São Paulo, Brazil (Protocol CEUA-P-20143), and was performed according to the National Institutes of Health guidelines for the use of experimental animals.

This study is part of an experimental sequence applied to ECMO-supported animals. Other aspects of these experiments have been published elsewhere.^(9,10)^ The instrumentation, surgical preparation, sepsis induction, and pulmonary injury were performed as previously described and published in this journal.^(11,12)^

**Figure 1:**
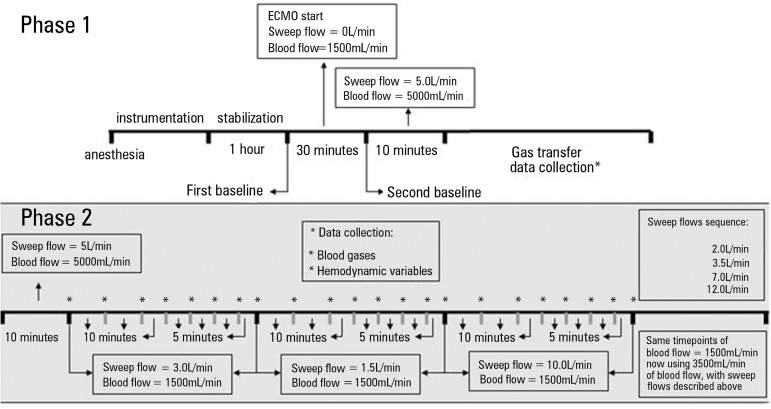
Timeline of the entire study. The gray ground illustrates the sequence of the current analysis. ECMO - extracorporeal membrane oxygenation. * Data collection.

The animal was maintained in apnea with 10cmH_2_O of positive end-expiratory pressure (PEEP) and an FiO_2_ = 1.0 using a concentric coil-resistor PEEP valve (Vital Signs Inc., Totowa, NJ) with a humidified continuous oxygen flow of 10.0L/min. ECMO blood flow was initially set to 5.0L/min, and the sweep flow was set to 5.0L/min. After 10 minutes, clinical and laboratory data were collected, and the blood flow was reduced to 1500mL/min, with an initial sweep flow = 3.0L/min. An arterial blood sample was collected every 10 minutes for up to 30 minutes. Subsequently, an arterial blood sample was collected every 5 minutes until 50 minutes after applying the new ECMO settings. Hemodynamic data were also collected with the blood samples. After this step, blood flow was maintained at 1500mL/min, and the sweep flow was set to 1.5L/min and, subsequently, to 10.0L/min at 50-minute intervals. The blood flow was then increased to 3500mL/min, and a sequence of sweep flows of 2.0, 3.5, 7.0 and 12.0L/min of 50 minutes each was applied. The same 50-minute data collection sequence described (for blood flow = 1500mL/min and sweep flow = 3.0L/min) was performed for each new tested sweep gas flow tested (Figure 1).

The following data were collected every ten minutes: heart rate (HR), mean arterial blood pressure (ABPm), central venous pressure, mean pulmonary artery pressure, pulmonary artery occlusion pressure, cardiac output, core temperature, peripheral oxygen saturation, end-tidal CO_2_ (EtCO_2_), and mixed venous oxygen saturation (SvO_2_). Pre- and post-membrane port blood samples from the pulmonary and femoral arteries were collected after ten minutes of each new tested sweep flow. Subsequently, a new femoral artery blood sample was collected every ten minutes for up to 30 minutes and every five minutes for up to 50 minutes at the beginning of each new sweep flow tested thereafter. Blood samples were analyzed in a standard radiometer ABL 600 (Radiometer, Copenhagen, Denmark). Biochemical samples were collected from the femoral artery catheter.

Calculations were performed using standard formulas as follows:^(13-16)^

- Blood oxygen content C_b_O_2_ [mL O_2_/100mL blood] = 1.36 x Hb x Sat_b_O_2_ + 0.0031 x P_b_O_2_
- Alveolar O_2_ pressure = capillary O_2_ partial pressure = PaO_2_ = (FiO_2_ x (694 - 46)) - (PaCO_2_/0.8)
- Capillary O_2_ saturation = 100%
- Pulmonary shunt [mL O_2_/100mL blood] = (capillary O_2_ content - arterial O_2_ content)/(capillary O_2_ content - venous O_2_ content)
- Blood CO_2_ content [mL/min] = ((1 - ((0.0289 x Hb)/(3.352 - 0.456 x (SatO_2_ /100) x (8.142 - pH)))) x 2.226 x 0.0307 + (0.00057 x (37 - temperature)) + (0.00002 x (37 - temperature)^2^) x PaCO_2_ x (1 + 10 ^(pH - 6.086 + (0.042 x (7.4 - pH)^) + ((38 - temperature) x 0.00472 + (0.00139 x (7.4 - pH)))))

### Statistical analysis

Data normality was assessed with the Shapiro-Wilk goodness-of-fit model. Normal data are presented as the means ± the standard deviations, and non-normal data are presented as the median and the 25^th^ and 75^th^ percentiles. Within group comparisons were performed using Friedman's test. Multivariate analysis was performed using a backward elimination mixed linear generalized model with the animals as random factors to account for the within-subject correlation among the repeated observations. The Markov chain Monte Carlo procedure with 10,000 simulations to reach the equilibrium of distributions was used to retrieve a fixed probability of each resulting independent variable from the mixed generalized model after backward elimination. Collinearity among the independent variables was tested with the Spearman's test of correlations in a matrix that included all of the tested variables. Variables with r coefficients > 0.85 were further tested for multicollinearity with the variance inflation factor (VIF). Variables with VIFs < 2.5 were considered appropriate for the analysis. The pseudo-R^2^ was calculated for each model to determine the goodness of fit. This calculation was performed with the squared ratio of the Spearman correlation between the fitted values of the model and the original values retrieved from the experiment. The R free source statistical package and comprehensive-R archive network (CRAN)-specific libraries were used to create the graphics and analyze the data.^(17)^

## RESULTS

In four animals, 20-French catheters were used, and a 21-French catheter was used in one animal to drain the blood into the ECMO device. In three animals, 21-French return catheters from the ECMO system were used, and 20-French catheters were used in two. To ensure non-significant re-circulation, the pre-membrane oxygen saturation was collected ten minutes after the beginning of each sweep gas flow test. The values obtained were 58% [51, 67] at a blood flow of 1,500mL/minute and 65% [64; 70] at a blood flow of 3,500mL/minute. There was no need to re-position the cannulae.

**Figure 2:**
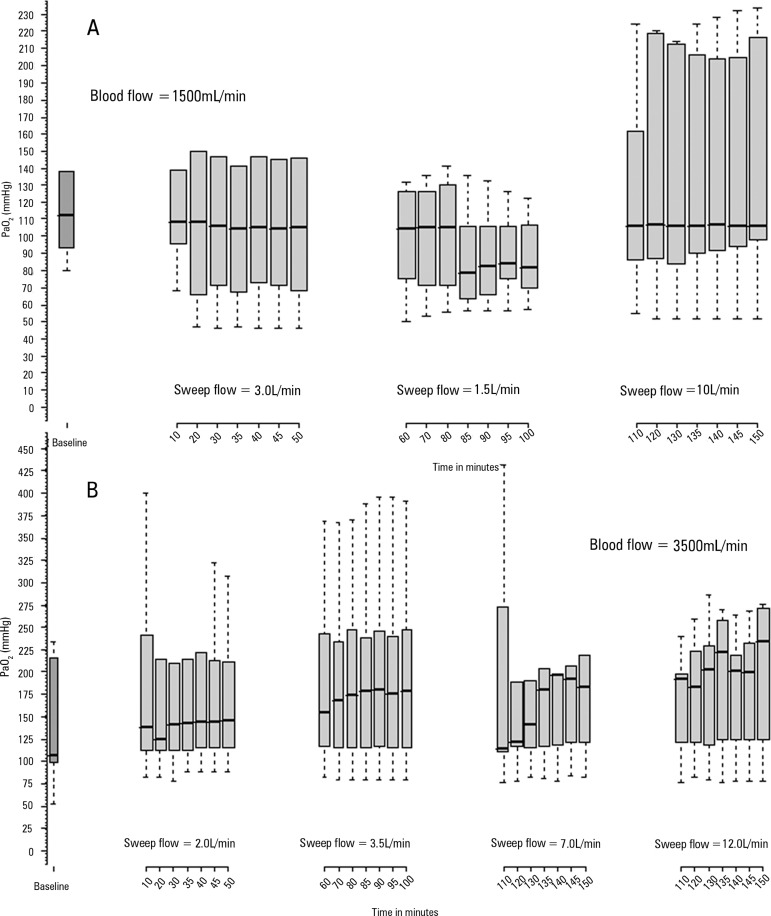
Observations of O_2_ partial pressure behaviors up to 50 minutes after sweep gas flow variations. A) Variations during the blood flow set at 1500mL/minute. At baseline, the blood flow was 5000mL/min, and the sweep flow was 5.0L/min; B) O_2_ partial pressure with the extracorporeal membrane oxygenation blood flow set at 3500mL/min. At baseline, the blood flow was 1500 mL/min, and the sweep flow was 10L/min. PaO_2_ - partial pressure of oxygen.

Figure 2 illustrates blood PaO_2_ behavior during the 50 minutes of observation. Among the other variables tested in the multivariate analysis, the variations in the experimental parameters were as follows: the pulmonary shunting (%) ranged from 45 [29, 66] to 60 [33, 67]; the temperature (°C) ranged 37.2 [36.8, 38.5] to 38.0 [37.0, 38.1]; the hemoglobin (g/dL) ranged from 11 [10, 13] to 14 [13, 14]; the cardiac output (L/min) ranged from 6.3 [4.0, 6.7] to 9.0 [4.4, 9.1]; the pH ranged from 7.03 [7.01, 7.16] to 7.43 [7.37, 7.49]; and the blood CO_2_ content (mL/100mL) ranged from 58 [57, 64] to 67 [63, 74].

**Figure 3:**
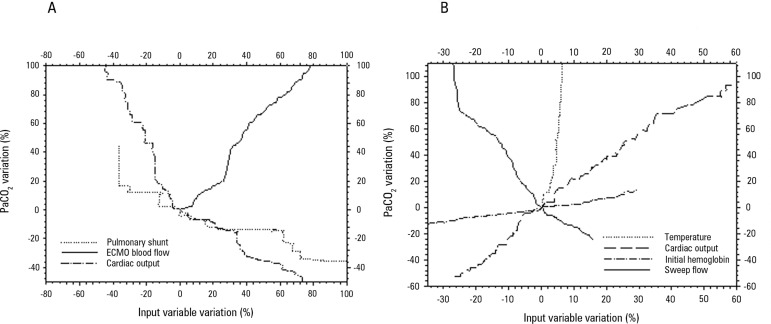
Spider plots based on the data collected from the animals illustrating the PaO_2_ (Panel A) and PaCO_2_ percent (Panel B) variations associated with the variations in the main related variables. The respective main related variables were extracted from the multivariate analysis. PaO_2_ - partial pressure of oxygen; PaCO_2_ - partial pressure of carbon dioxide.

Table 1 presents the multivariate analysis evaluating the factors associated with blood PaO_2_ and PaCO_2_ after 50 minutes of ECMO blood and sweep gas flows. Figure 3 displays a spider plot quantifying the influences of the deviation of each variable extracted from the multivariate analysis on the final (after 50 minutes) PaO_2_ and PaCO_2_. Table 2 presents the hemodynamic behavior during the study.

**Table 1:** Backward elimination multivariate analysis exploring the variables associated with plasma oxygen and carbon dioxide partial pressures during extracorporeal membrane oxygenation support

**Variable**	**Beta-unstandardized coefficient**	**p value**	**VIF**
Plasmatic oxygen partial pressure analysis			
Blood flow (1mL/minute)	0.036	< 0.001	1.28
Cardiac output (1mL/minute)	-11.970	< 0.001	1.73
Pulmonary shunt (1%)	-0.232	< 0.001	1.35
Initial PaO_2_ (1mmHg)	0.160	< 0.001	1.65
Initial PaCO_2_ (1mmHg)	0.442	0.01	1.41
Plasmatic carbon dioxide partial pressure analysis			
Cardiac output (1mL/minute)	3.578	< 0.001	2.41
Sweep (1L/minute)	-2.635	< 0.001	1.18
Temperature (ºC)	4.514	< 0.001	2.16
Initial pH (0.01)	-66.065	< 0.001	1.40
Hemoglobin (1g/dL)	6.635	< 0.001	2.10

This multivariate analysis was performed using a mixed model with backward elimination. The initial dependent variables in the systemic oxygenation analysis were blood flow, cardiac output, pulmonary shunting, initial PaO_2_, initial PaCO_2_, temperature and hemoglobin. Temperature and hemoglobin were removed during the backward elimination of the multivariate analysis. The coefficient of determination of the final model (pseudo - R^2^) was 0.61. The initial dependent variables in the carbon dioxide transfer analysis were sweep flow, cardiac output, temperature, initial pH and hemoglobin. The coefficient of determination of the final model (pseudo - R^2^) was 0.79. Blood samples were acquired from the pre-membrane port. Beta-unstandardized coefficient - estimated variation in the oxygen transference in mL/min for each unit (the units are cited in the table) variation in the independent variables. VIF - variance inflation factor; PaO_2_ - partial pressure of oxygen; PaCO_2_ - partial pressure of carbon dioxide.

**Table 2:** Hemodynamic variables after fifty minutes of sweep or blood flow modifications

**Variable**	**Blood flow = 1,500mL/min**				**Blood flow = 3,500mL/min**			p value*
	**Sweep flow 3.0L/min**	**Sweep flow 1.5L/min**	**Sweep flow 10L/min**	**Sweep flow 2.0L/min**	**Sweep flow 3.5L/min**	**Sweep flow 7.0L/min**	**Sweep flow 12L/min**	
Cardiac output (L/min)	6.5 [6.4, 6.9]	6.3 [4.0, 6.7]	7.7 [3.6, 9.4]	7.3 [4.3, 9.3]	9.0 [4.4, 9.1]	7.6 [4.8, 8.2]	6.3 [4.8, 7.9]	0.834
Heart rate (bpm)	134 [124, 145]	133 [95, 137]	135 [123, 147]	128 [124, 149]	137 [131, 160]	121 [121, 153]	128 [123, 161]	0.867
ABPm (mmHg)	126 [106, 126]	106 [100,125]	101 [91, 112]	98 [92, 105]	103 [94, 114]	113 [112, 122]	122 [119, 123]	0.154
PAPm (mmHg)	54 [41, 59]	46 [42, 47]	36 [32, 46]	47 [34, 48]	40 [31, 47]	42 [35, 45]	39 [33, 44]	0.248
CVP (mmHg)	6 [5, 13]	6 [4, 16]	5 [4, 13]	5 [3, 15]	5 [4, 14]	5 [4, 11]	5 [4, 11]	0.299
PAOP (mmHg)	11 [10, 12]	8 [8, 11]	8 [7, 11]	7 [5, 9]	8 [7, 8]	8 [6, 16]	8 [7, 17]	0.715

ABPm - mean arterial blood pressure; PAPm - mean pulmonary arterial blood pressure; CVP - central venous pressure; PAOP - pulmonary artery occlusion pressure.

* The p values were obtained based on Friedman’s tests. Post-hoc analyses were not performed due to the large variety of comparisons (varying through the blood flow and sweep gas flows domains).

## DISCUSSION

After changing the blood flow, the oxygen partial pressure reached equilibrium almost immediately, and variations in the sweep gas flow did not improve oxygenation. Oxygenation after 50 minutes was mainly associated with ECMO blood flow (beta coefficient = 0.036mmHg/mL/min), cardiac output (beta coefficient = -11.970mmHg/L/min), and pulmonary shunting (beta coefficient = -0.232mmHg/%). Furthermore, the initial PaO_2_ and PaCO_2_ were also associated with blood oxygenation, with beta coefficients of 0.160 and 0.442mmHg/mmHg of the given gas, respectively. PaCO_2_ after 50 minutes was associated with cardiac output (beta coefficient = 3.578mmHg/L/min), sweep gas flow (beta coefficient = -2.635mmHg/L/min), temperature (beta coefficient = 4.514 mmHg/°C), initial pH (beta coefficient = -66.065mmHg/0.01 unit) and hemoglobin (beta coefficient = 6.635mmHg/g/dL).

Compared with carbon dioxide, the volume of the distribution of oxygen in the body is small. This is in line with our finding of a very fast oxygen equilibrium after a step change in the ECMO settings and contrasts with the long time required to reach PaCO_2_ equilibrium; however, this issue should be the subject of another manuscript due to its complexity.

It is interesting to consider the physiological bases of the factors associated with the final arterial PaO_2_ at equilibrium. The dependence of arterial oxygenation on ECMO blood flow occurred primarily because only a fraction of the cardiac output was oxygenated by the veno-venous ECMO. The remaining fraction of the venous return crossed the vena cava native bed straight to the heart without gas exchange and thus shunted the ECMO circuit.^(8)^ With higher ECMO blood flows for a given venous return, this fraction increases, which leads to increased oxygen delivery by the return cannula. This increased delivery occurs despite the fall in the PaO_2_ of the return cannula that results from the limited diffusibility of oxygen through the lung membrane.^(18)^ Additionally, the lungs, however sick, provide an additive oxygenation effect (in series with the ECMO circuit). The delivery of highly oxygenated blood to the lungs (from the ECMO circuit) may worsen their ventilation perfusion mismatch by impeding hypoxic vasoconstriction. Despite these considerations, our finding that arterial oxygenation increases with increasing ECMO blood flow implies that the fall in oxygen content and the worsening of hypoxic vasoconstriction are offset by the increase in blood flow. Notably, the reverse of the above described effect, i.e., the reduction of cardiac output (for example, due to the use of beta-blockers) has been described to optimize peripheral oxygenation during veno-venous ECMO support.^(19)^

The pre-ECMO PaCO_2_ value was also related to oxygenation, most likely due to modulation of hemoglobin-oxygen affinity.^(20)^ Finally, in an intuitive manner, the lower pre-ECMO PaO_2_ is also associated with greater oxygenation. In a recent study, Messai et al. demonstrated that peripheral oxygen saturation can be predicted based on ECMO blood flow, cardiac output, after-membrane oxygen saturation, and pulmonary artery oxygen saturation.^(3)^ These findings are very similar to those of the current study; however, the importance of the pulmonary shunt was highlighted in this experiment.

The following five variables were associated with the PaCO_2_. (1) The cardiac output is the main determinant of the local tissue flow and therefore is a modulator of tissue CO_2_ transport to the core circulation and to the lungs.^(21)^ Increased cardiac output results in enhanced CO_2_ transportation from the peripheral tissues to the lungs. In contrast, an elevation of the PaCO_2_, regardless of the cause, initiates a sympathetic autonomic response and, consequently, an elevation in cardiac output.^(22)^ In this experiment, it is difficult to ascribe cause and consequence. (2) Sweep flow is an intuitive PaCO_2_ modulator once the membrane is ventilated by the gas flow.^(6)^ (3) Temperature is an aerobic metabolism regulator that leads to increased or decreased CO_2_ production.^(23)^ (4) Initial blood pH is also a PaCO_2_ modulator because it can disturb the transportation, storage, and production of CO_2_.^(23-25)^ (5) Hemoglobin is a PaCO_2_ regulator because an increased hemoglobin level represents increased CO_2_, resulting in a lower partial pressure of CO_2_ in the plasma.^(25)^

In a bedside practical approach, taking PaO_2_ and PaCO_2_ together, ECMO blood flow and sweep gas flow are important variables for ECMO support.^(6)^ Furthermore, in special clinical situations, such as persistent hypoxemia and/or hypercapnia, patient temperature and cardiac output are variables that exhibit strong interactions and strong effects on oxygenation and CO_2_ removal, which can be modulated by the care team. The hemoglobin level is a particularly important variable in ECMO-supported patients. Higher hemoglobin levels are associated with increased oxygen transport,^(26)^ and according to our findings, associated with lower blood PaCO_2_. Currently, some ECMO groups allow hemoglobin levels as low as 7g/dL.^(27)^ However, experienced groups keep hemoglobin levels above 10g/dL, which results in high survival rates for severely hypoxemic patients.^(7)^

The hemodynamics of animals exhibited low sweep flows that were associated with increased pulmonary artery pressure and reduced cardiac output. In addition to these findings, a higher mean systemic arterial pressure was also observed, which likely resulted from sympathetic activation.

In this manuscript, we described the associations of some variables with the oxygenation and final PaCO_2_ of the ECMO-supported patient. However, we would like to stress that other classical variables, such as the sweep gas flow FiO_2_, subject weight and type and surface area of the oxygenator, are also associated with gas transfer and the consequent final blood gases.^(27)^

This study has some limitations. First, the low number of animals used may have resulted in type II errors that attenuated the validity of our findings regarding the lack of associations. Notably, this limitation would not affect our positive findings. Second, these results are based on an animal model with a physiology different from that of humans.

## CONCLUSION

In conclusion, elevations in the blood and sweep gas flows in an apneic veno-venous extracorporeal membrane oxygenation model resulted in increased partial oxygen and reduced partial carbon dioxide pressures. Furthermore, without the possibility of causal inference, the partial pressure of oxygen was negatively associated with pulmonary shunting and cardiac output, and the partial pressure of carbon dioxide was positively associated with cardiac output, core temperature and initial hemoglobin.
